# Protective effect of DHEA on hydrogen peroxide-induced oxidative damage and apoptosis in primary rat Leydig cells

**DOI:** 10.18632/oncotarget.15300

**Published:** 2017-02-13

**Authors:** Xiao Ding, Lei Yu, Chongyang Ge, Haitian Ma

**Affiliations:** ^1^ Key Laboratory of Animal Physiology and Biochemistry, College of Veterinary Medicine, Nanjing Agricultural University, Nanjing, China

**Keywords:** dehydroepiandrosterone, oxidative damage, apoptosis, Leydig cells, Pathology Section

## Abstract

Dehydroepiandrosterone (DHEA) is widely used as a nutritional supplement due to its putative anti-aging properties. However, the effect of DHEA in Leydig cells, a major target cell of DHEA biotransformation in male, are not clear. The present study aimed to investigate the preventative effect of DHEA on oxidative damage and apoptosis after H_2_O_2_ treatment in Leydig cells. The results showed that DHEA treatment attenuated the reduction of cell viability induced by H_2_O_2_. No differences were observed on the superoxide anion (O_2_^-^) content, while DHEA treatment decreased reactive oxygen species (ROS) and hydroxyl radical (OH) content in H_2_O_2_-treated Leydig cells. Pre-treatment with DHEA increased peroxidase (POD) activity and decreased glutathione peroxidase (GSH-Px) activity in H_2_O_2_-treated Leydig cell. DHEA treatment attenuated DNA damage as indicated by the decreasing of tail moment, comet length and olive tail moment. Total apoptosis ratio and early apoptosis ratio were significantly decreased in H_2_O_2_-treated Leydig cell that were pre-treatment with DHEA. DHEA treatment decreased *Bax*, *capase-9* and *capase-3* mRNA levels in H_2_O_2_-treated Leydig cells. Our results demonstrated that pre-treatment with DHEA prevented the Leydig cells oxidative damage caused by H_2_O_2_ through increasing POD activity, which resulted in inhibition of OH generation. Meanwhile, pre-treatment with DHEA inhibited H_2_O_2_-induced Leydig cells early apoptosis which mainly by reducing the pro-apoptotic protein *Bax* and *caspases-9*, *caspases-3* mRNA levels. This information is important to understand the molecular mechanism of anti-ageing effect and potential application in treatment of oxidative stress induced related diseases of DHEA.

## INTRODUCTION

Dehydroepiandrosterone (DHEA), an important precursor of activate steroid hormone, is produced abundantly by the adrenal cortex with an age-dependent pattern [1]. Age-dependent decrease of DHEA production has attracted extensive attention due to its possible benefit to psychological well-being in the elderly [2]. The literature evidences reveal the changes in DHEA level are associated with multiple pathologies, whereas a replacement therapy might alleviate age-associated declines in physiological functions [3–7]. This results in a large-scale self-administration of DHEA as an “anti-ageing” drug in dietary supplement. Many studies had reported that DHEA had antioxidant effects in various acute and chronic oxidative stress experiments [7–10]. However, the effects of DHEA administration can be antioxidant [11] or pro-oxidant [12, 13], depending on the administered dose and specific tissue [14]. Gallo *et al*. reported that slightly higher concentrations of DHEA protected cells against lipid peroxidation induced by oxidative stress while pharmacological doses of DHEA displayed a pro-oxidant activity [15]. In addition, microsomes isolated from DHEA-treated rats are resistant to iron-dependent lipid peroxidation, whereas microsomes pre-incubated with DHEA *in vitro* are not resistant, suggesting that a cell or tissue dependent signaling or transformation is required for DHEA’s antioxidant action [16].

Aging results in the progressive deterioration of physiological function, and free radical theory is the most accepted ageing theories [17]. In aging process, reactive oxygen species (ROS), can be generated; reactive oxygen species is toxic at high concentrations [17]. ROS can interact with many molecules which will result in DNA mutation, protein denaturation, lipid peroxidation, membrane destruction and so on [18]. Furthermore, excess ROS can activate apoptotic pathways [19]; a biochemical hallmark of apoptosis is DNA damage [20]. Administration of antioxidants attenuates free radical-mediated oxidative damage in several organs including the testis [21]. Oxidative stress plays a key role in cell damage [22] and the risk of oxidative damage is especially high for steroid synthesizing tissues, which use molecular oxygen for steroids biosynthesis [23]. The study shows that DHEA exerts its effects by rapidly transforming into biologically active steroids in target tissues [24]. It had proposed that increasing serum DHEA (60-79 years old) concentration to the levels found in young people may have anti-ageing effects [1]. Our previous study demonstrated that administration of DHEA markedly increased serum testosterone concentration in rats [25]. In males, ∼95% of androgen biosynthesis and secretion occurs in Leydig cells, and it had been certified that functional changes in Leydig cells is account for the observed reduction in serum testosterone level [2].

Taken these points together, we presumed that DHEA protects cell from oxidative damage, which might be a major reason for the anti-ageing action of DHEA. In addition, the effect of DHEA on the antioxidant function of Leydig cells, a major target cell of DHEA convert to active steroids, is unknown. Thus, the present study aimed to investigate the effect of DHEA on ROS generation, antioxidant enzymes activity, DNA damage, cell apoptosis and apoptosis-related factors in H_2_O_2_-treated rat Leydig cells, and this information is important to understand the molecular mechanism of anti-ageing action of DHEA.

## RESULTS

### Protective effect of DHEA on cell viability

**Figure 1:**
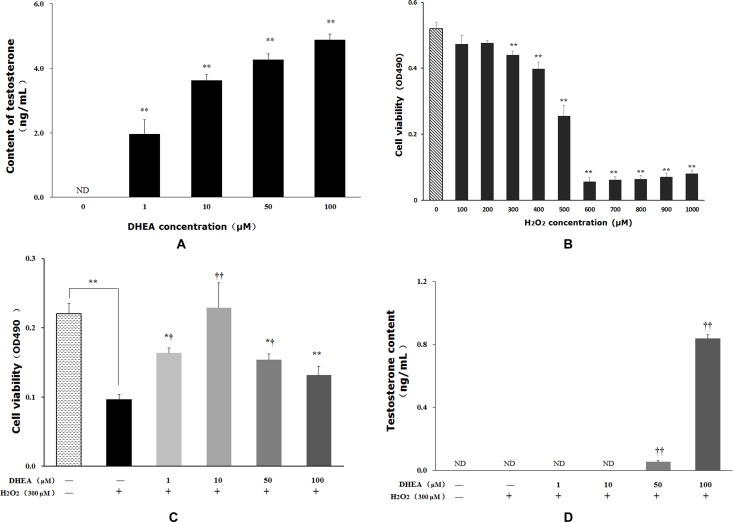
Impact of DHEA on testosterone content and cell viability in Leydig cells Data are presented as means ± SD from three independent experiments, with six samples taken at each treatment group (*n* = 6). **A**. Testosterone content in Leydig cell with DHEA-treated; **B**. Cell viability in Leydig cell with H_2_O_2_-induced; **C**. Effect of DHEA-pretreated on cell viability in H_2_O_2_-induced Leydig cells; **D**. Effect of DHEA-pretreated on testosterone content in H_2_O_2_-induced Leydig cells. ND = none detected. *P < 0.05 and ** *P* < 0.01, relative to DHEA and H_2_O_2_-free control; ^†^ *P* < 0.05 and ^††^ *P* < 0.01, relative to H_2_O_2_-induced control.

Testosterone content was significantly increased in primary Leydig cells after DHEA treatment (*P* < 0.01) (Figure 1A). Treated with H_2_O_2_ reduced cell viability in a dose-dependent manner, and 300μM H_2_O_2_ treatment significantly decreased cell viability relative to that in H_2_O_2_-free group (Figure 1B). Pre-treatment with 1-50μM DHEA significantly enhanced cell viability (*P* < 0.05) (Figure 1C). Meanwhile, 50-100μM DHEA significantly increased testosterone content when compared to H_2_O_2_-treated group (*P* < 0.01) (Figure 1D).

### DHEA inhibit reactive oxygen species generation

**Figure 2:**
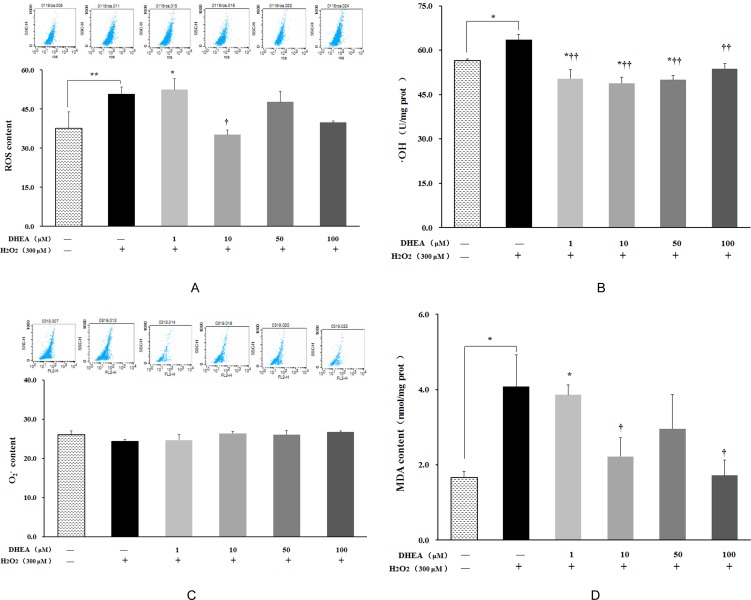
Impact of DHEA on oxidant generation and malondialdehyde content in H_2_O_2_-induced Leydig cells Data are presented as means ± SD from three independent experiments, with six samples taken at each treatment group (*n* = 6). **A**. Reactive oxygen species (ROS) content; **B**. Superoxide anion (O_2_^-^); content **C**. Hydroxyl Radical (·OH) content; **D**. Malondialdehyde (MDA) content. *P < 0.05 and ** *P* < 0.01, relative to DHEA and H_2_O_2_-free control; ^†^ *P* < 0.05 and ^††^ *P* < 0.01, relative to H_2_O_2_-induced control.

ROS, ·OH and MDA contents were significantly increased in H_2_O_2_-treated group when compared to control group (*P* < 0.05) (Figure 2). Pre-treatment with 10μM DHEA reduced intracellular ROS levels relative to that in H_2_O_2_-treated group (*P* < 0.05) (Figure 2A). Pre-treatment with 1-100μM DHEA significantly decreased ·OH content (*P* < 0.01) (Figure 2B), while no significant differences were observed on the O_2_^-^ content (Figure 2C). Compared to H_2_O_2_-treated group, MAD contents were decreased in the cells pre-treated with 10 and100μM DHEA (*P* < 0.05) (Figure 2D).

### Impact of DHEA on antioxidant enzyme activity

**Figure 3:**
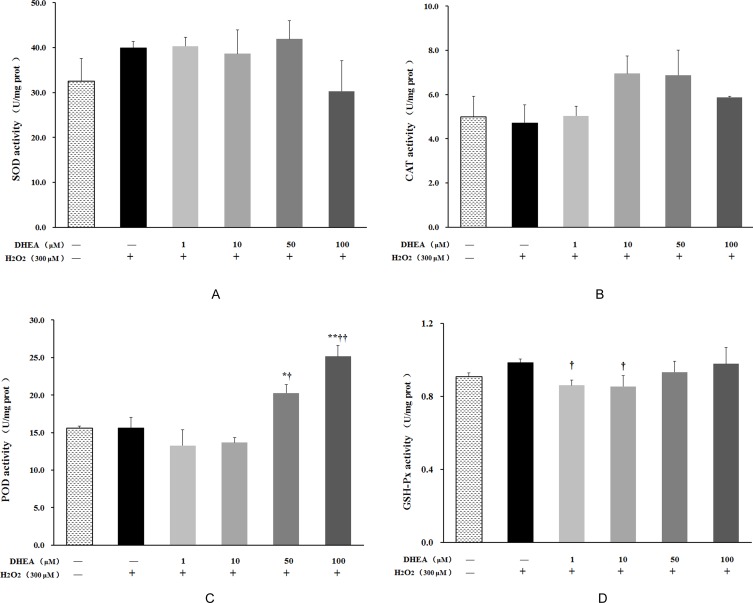
Effect of DHEA on antioxidant enzyme activity in H_2_O_2_-induced Leydig cells Data are presented as means ± SD from three independent experiments, with six samples taken at each treatment group (*n* = 6). *P < 0.05 and ** *P* < 0.01, relative to DHEA and H_2_O_2_-free control; ^†^ *P* < 0.05 and ^††^ *P* < 0.01, relative to H_2_O_2_-induced control. SOD = superoxide dismutase; CAT = catalase; POD = peroxidase; GSH-Px = glutathione peroxidase.

As shown in Figure 3, no changes were observed on SOD, GSH-Px, CAT or POD activities in H_2_O_2_-treated group (*P*>0.05). Pre-treatment with 50μM and 100μM DHEA significantly increased POD activity when compared to H_2_O_2_-treated group (*P* < 0.05) (Figure 3C), while 1μM and 10μM DHEA significantly decreased GSH-Px activity relative to that in H_2_O_2_-treated group (*P* < 0.05) (Figure 3D). Compared to H_2_O_2_-treated group, pre-treatment with different dose DHEA have no significant effect on SOD and CAT activities (*P*>0.05) (Figure 3A and 3B).

### Protective effect of DHEA on H_2_O_2_ -induced oxidation damage

**Figure 4:**
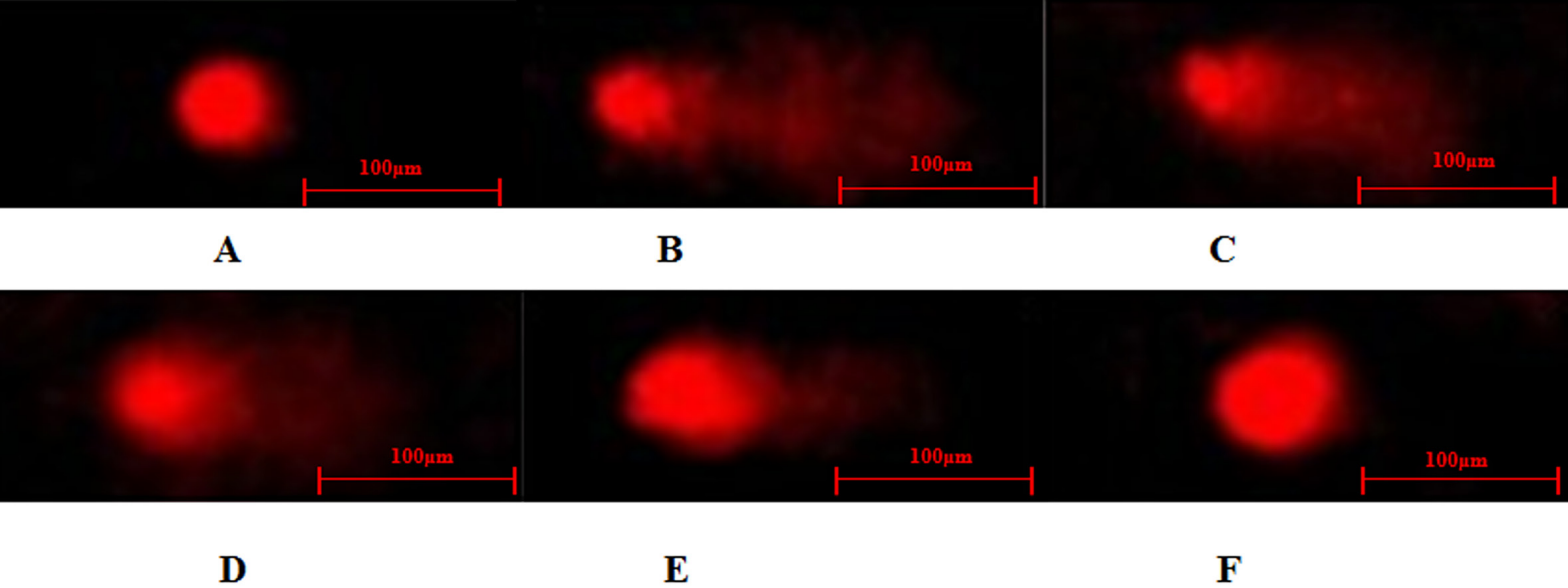
Typical DNA comet images of Leydig cells treated with DHEA and H_2_O_2_ **A**. control group; **B**. H_2_O_2_-induced control; **C**.-**F**. 1-100μM DHEA treatment group with 300μM H_2_O_2_-induced oxidation damage.

The comet assay on DNA damage in H_2_O_2_-induced Leydig cells was shown in Figure 4 and Table 2. Exposure of Leydig cells to H_2_O_2_ caused a significant increase in DNA damage, as indicated by the greater migration of DNA fragments on the agarose gel (Figure 4). Comet length, tail moment and olive tail moment were significantly higher in cells exposed to 300μM H_2_O_2_ when compared to the H_2_O_2_-free control (*P* < 0.05) (Table 2). Pre-treatment with DHEA prevented H_2_O_2_-induced DNA damage in a dose-dependent pattern (Figure 4). As shown in Table 2, pre-treatment with 50-100μM DHEA significantly decreased comet length relative to that in H_2_O_2_-treated group (*P* < 0.01). When compared to H_2_O_2_-treated group, tail moment and olive tail moment were significant decreased in 10-100μM DHEA-pretreated group (*P* < 0.01).

**Table 2:** Effect of DHEA on the H_2_O_2_-induced DNA damage reflecting as different comet parameters in Leydig cell

Comet parameters	Comet length (μm)	Tail moment (μm)	Olive tail moment (μm)	Head DNA (%)	Tail DNA (%)
control	69.25±5.08	13.18±1.89	3.42±0.80	72.43±4.31	27.57±4.31
300μM H_2_O_2_	102.79±8.06 *	50.16±7.08 **	18.61±4.30**	59.43±3.77*	40.57±3.77*
1μM DHEA+ 300μM H_2_O_2_	118.11±15.18**	49.88±9.88**	15.63±3.92*	65.45±2.97	34.55±2.97
10μM DHEA+ 300μM H_2_O_2_	98.00±8.85	21.59±3.64^††^	6.81±1.59^††^	63.02±3.60	36.99±3.60
50μM DHEA+ 300μM H_2_O_2_	70.48±4.68 ^††^	22.67±3.00^††^	4.32±0.47^††^	65.63±4.54	34.37±4.54
100μM DHEA+ 300μM H_2_O_2_	64.00±2.56^††^	6.16±1.08^††^	0.68±0.14^††^	90.34±1.18^††^	29.66±1.18^††^

Data are presented as means±SD from three individual experiments, with six samples taken at each time point (*n* = 6). *P < 0.05 and **P < 0.01, relative to DHEA and-H_2_O_2_-free control; ^†^*P* < 0.05 and ^††^*P* < 0.01, relative to H_2_O_2_-induced control.

### DHEA reduced H_2_O_2_-induced cells apoptosis

**Figure 5:**
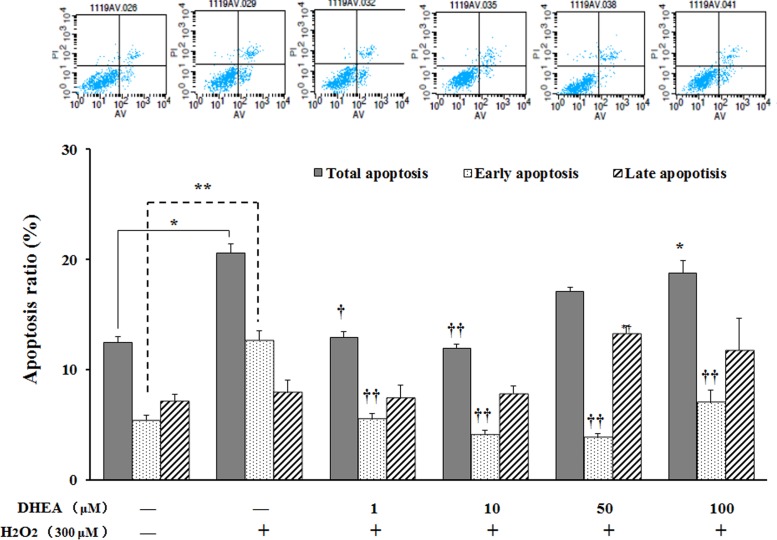
Effect of DHEA on cells apoptosis in H_2_O_2_-induced Leydig cells Data are presented as means ± SD from three independent experiments, with six samples taken at each treatment group (*n* = 6). *P < 0.05 and ** *P* < 0.01, relative to DHEA and H_2_O_2_-free control; ^†^ *P* < 0.05 and ^††^ *P* < 0.01, relative to H_2_O_2_-induced control.

No differences were observed on late apoptosis ratio, while total apoptosis ratio and early apoptosis ratio were significantly increased in H_2_O_2_-treated group when compared to the H_2_O_2_-free control (*P* < 0.05) (Figure 5). Pre-treatment with 1-10μM DHEA significantly decreased total apoptosis ratio (*P* < 0.05) and 1-100μM DHEA significantly decreased early apoptosis ratio when compared to H_2_O_2_-treated group (*P* < 0.01), while no differences were observed on the late apoptosis ratio (*P*>0.05) (Figure 5).

### Effect of DHEA on the mRNA expression levels of apoptosis related factors

**Figure 6:**
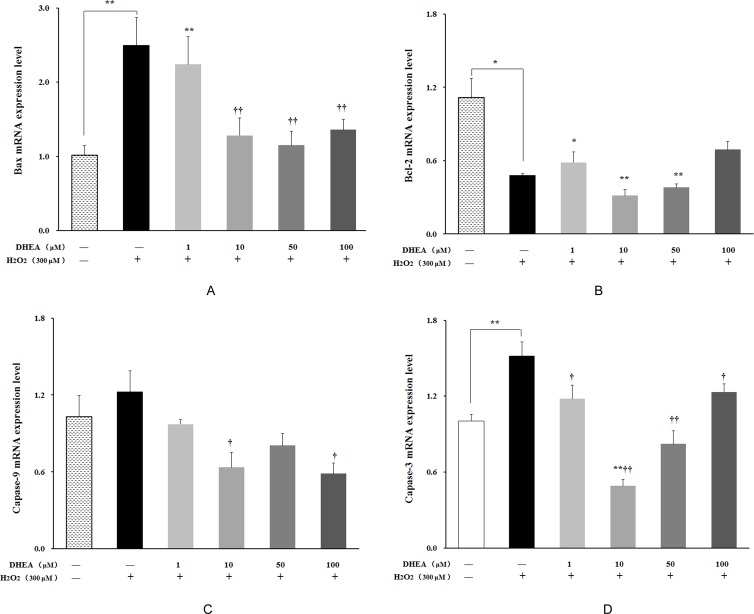
Effect of DHEA on *Bax*, *Bcl-2*, *Capase-9* and *Capase-3* mRNA levels in H_2_O_2_-induced Leydig cells Data are presented as means ± SD from three independent experiments, with six samples taken at each treatment group (*n* = 6). **A**. *Bax* mRNA level; **B**. *Bcl-2* mRNA level; **C**. *Capase-9* mRNA level; **D**. *Capase-3* mRNA level. *P < 0.05 and ** *P* < 0.01, relative to DHEA and H_2_O_2_-free control; ^†^ *P* < 0.05 and ^††^ *P* < 0.01, relative to H_2_O_2_-induced control.

As shown in Figure 6, *Bax* and *caspase-3* mRNA levels were significantly increased (*P* < 0.01), while *Bcl-2* mRNA level was significantly decreased in H_2_O_2_-treated group when compared to the H_2_O_2_-free control (*P* < 0.05). Pre-treatment with DHEA has no effect on *Bcl-2* mRNA level (*P*>0.05) (Figure 6B), while *Bax* and *capase-3* mRNA levels were significantly decreased in 10-100μM DHEA-pretreated group relative to that in H_2_O_2_-treated group (*P* < 0.05) (Figure 6A and 6D). Compared to H_2_O_2_-treated group, pre-treatment with 10μM and 100μM DHEA significantly decreased *capase-9* mRNA level (*P* < 0.05) (Figure 6C).

## DISCUSSION

In aging individuals, lipid peroxidation, oxidative stress and damage of cellular macromolecules caused by the excessive reactive oxygen species has been hypothesized to be one of the major contributors to the aging process [23]. As one of the most important ROS, hydrogen peroxide (H_2_O_2_) has been extensively used to induce oxidative stress *in vitro* models [28]. Our results showed that the cell viability significantly decreased in 300-1000μM H_2_O_2_-treated groups than that in H2O2-free group. Previous study had reported that exposure to low doses of H_2_O_2_ induces apoptosis in a variety of cell types, thereby directly establishing oxidative stress as a mediator of cell death [29, 30]. The high doses of H_2_O_2_ have been shown to be cytotoxic by triggering a disorderly form of cell death, or necrosis [30, 31]. Thus, we used 300μM H_2_O_2_ treatment in the subsequent experiments, and the results showed that H_2_O_2_ significantly increased ROS content and total apoptosis ratio in Leydig cells. Meanwhile, the comet assay showed that Leydig cells exposed to 300μM H_2_O_2_ exhibited a significant increase in comet length, tail moment and olive tail moment. These results indicated that H_2_O_2_ caused oxidative stress, providing a model for further investigation the protective effect of DHEA on H_2_O_2_-induced oxidative damage in Leydig cells.

Previous studies had reported that administration of DHEA produce a number of beneficial effects in elderly [1–4, 6]. This study found that pre-treatment with DHEA decreased the ROS content by inhibiting ·OH generation and prevented cell death induced by H_2_O_2_. This is consistent with the results of Gabriel *et al*. [10], who reported that DHEA reduced ROS content in ovariectomized rats. Oxidative stress defined as excessive production of ROS include O_2_-, H_2_O_2_ and ·OH, and excessive ROS is toxic to human body [17]. Many recent evidences indicate that endogenous free radicals contribute to spontaneous mutagenesis by directly inducing DNA damage [32]. Pre-treatment with DHEA prevented H_2_O_2_-induced DNA damage in a dose-dependent pattern in Leydig cells, as suggested by the decrease of comet length, tail moment and olive tail moment in DHEA-treated groups. Based on the data above, we presumed that DHEA might prevent DNA oxidative damage in H_2_O_2_-induced Leydig cells by inhibiting ·OH generation.

It is well-known that body utilizes non-enzymatic and enzymatic antioxidants to avoid or to retard cellular damage arising from oxidative stress [10]. The antioxidant enzyme activity maintain the balance between the formation and scavenging of ROS [33, 34]. No changes were observed on the SOD and CAT activities, while pre-treatment with 50μM and 100μM DHEA significantly increased POD activity in H_2_O_2_-treated Leydig cells. DHEA supplementation demonstrated the antioxidant effects in various acute and chronic oxidative stress experiments in rodents [36]. It had reported that DHEA exhibits antioxidant properties by suppressing superoxide anion production [35]. Another antioxidant factor is the reduced glutathione (GSH), which act as a substrate in the detoxification of xenobiotic and in control of hydrogen peroxide and other peroxides concentrations [9]. Our results showed that 1μM and 10μM DHEA treatment decreased GSH-Px activity in H_2_O_2_-treated Leydig cells. NADPH is a cofactor required for the conversion of GSSG to GSH by glutathione reductase, and glucose-6-phosphate dehydrogenase (G6PDH) is an important enzyme involved in the production of NADPH [21]. *In vivo* and *in vitro* studies had shown that DHEA inhibits G6PDH activity which in turn reduced the production of NADPH [9, 21]. Thus, we speculated that DHEA decreased NADPH level through inhibiting G6PDH activity and finally decreased GSH-Px activity in H_2_O_2_-induced Leydig cells. These result indicated that pre-treatment with DHEA increased POD activity which results in the decrease of ROS contents in H_2_O_2_-treated Leydig cells.

Present study showed that pre-treatment with1μM and 10μM DHEA significantly decreased the total apoptosis ratio and early apoptosis ratio, but not the late apoptosis ratio. This result indicated that DHEA prevented H_2_O_2_-induced Leydig cells apoptosis and this action was achieved mainly through inhibiting early apoptosis. Permeabilization of the mitochondrial outer membrane allows the release of pro-apoptotic factors from intermembrane space into cytosol in early apoptosis [37]. Cytochrome c release can promotes and amplify the apoptotic cascade, which is considered as the commitment step of programmed cell death [37–39]. The Bcl-2 family proteins, including Bax and Bcl-2, are key regulators in the early stages of apoptotic [40–44]. Our results showed that pre-treatment with 10-100μM DHEA decreased the pro-apoptotic protein *Bax* mRNA level in H_2_O_2_-treated Leydig cells, while there is no effect on anti-apoptotic protein *Bcl-2* mRNA level. It well known that depending on the Bcl-2/Bax ratio, sequential activation of caspases-9 and caspases-3 plays a central role in the execution of cell apoptosis [45–47]. Our results also found that pre-treatment with 1-100μM DHEA significantly decreased the *caspases-3* mRNA levels in H_2_O_2_-treated Leydig cells. In addition, pre-treatment with10μM and 100μM DHEA treatment decreased the *caspases-9* mRNA levels in H_2_O_2_-treated Leydig cells. These results indicated that DHEA prevented H_2_O_2_-induced Leydig cells apoptosis mainly through inhibiting pro-apoptotic protein *Bax* mRNA levels which resulted in the decreasing of *caspases-9* and *caspases-3* mRNA levels.

**Figure 7:**
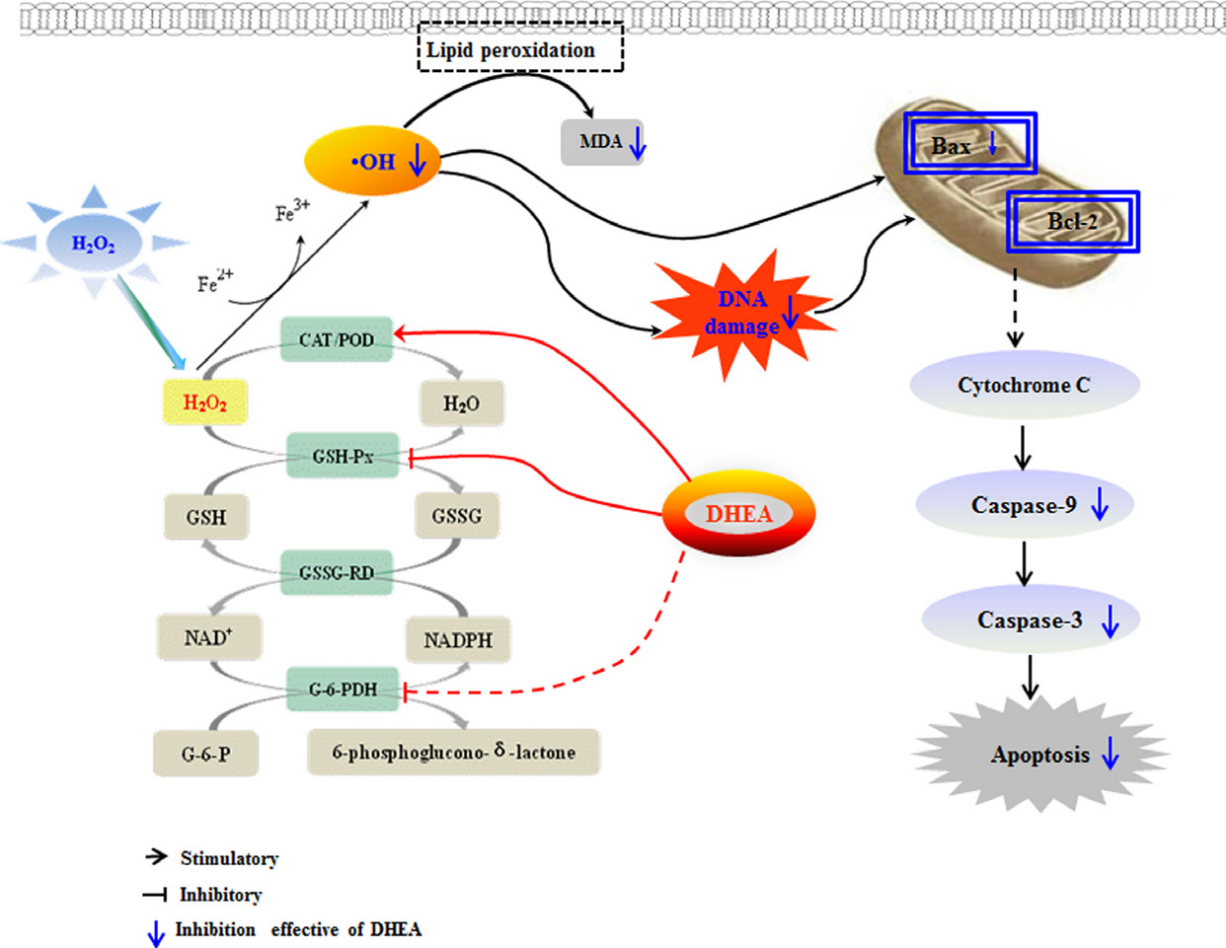
Mechanism of DHEA protective oxidation damage in H_2_O_2_-induced Leydig cells DHEA prevented H_2_O_2_-induced Leydig cells oxidative damage by increasing POD activity which resulted in the inhibiting ·OH generation. Meanwhile, DHEA decreased H_2_O_2_-induced cells apoptosis which mainly achieved through decrease the pro-apoptotic protein Bax, caspases-9 and caspases-3 mRNA levels.

In conclusion (as shown in Figure 7), our results demonstrated that pre-treatment with DHEA prevented H_2_O_2_-induced Leydig cells oxidative damage by increasing the POD activity which result in the inhibition of ·OH generation, and it decreased H_2_O_2_-induced Leydig cells apoptosis through decrease the pro-apoptotic protein *Bax* and *caspases-9*, *caspases-3* mRNA levels. This information shed light on the potential application for DHEA in elderly and treatment of oxidative stress induced related diseases. Certainly, further studies are warranted to better understand the underlying mechanisms on how DHEA prevents oxidative damage in the cells.

## MATERIALS AND METHODS

### Isolation of primary Leydig cells

Male Sprague-Dawley (SD) rats (200±20g) were purchased from the Shanghai Experimental Animal Center of the Chinese Academy of Sciences (Shanghai, China). Animals were housed under 25°C and 50% humidity with a 12-h light/dark cycle. The food and water were available *ad libitum*. All animal handling procedures were performed in strict accordance with guide for the Care and Use of Laboratory Animals central of the Nanjing Agricultural University. The protocol was approved by the Institutional Animal Care and Use Committee of Nanjing Agricultural University. All experiments were performed under sodium pentobarbital anesthesia, and all efforts were made to minimize suffering.

Leydig cells were isolated by enzymatic digestion and purified by a discontinuous Percoll gradient, as described by Murugesan *et al*. [23]. Briefly, testes were decapsulated with fine forceps without breaking the seminiferous tubules and digested in DMEM-F12 containing collagenase (0.5%) at 37°C for 15min in shaking water bath. After incubation, collagenase-free DMEM-F12 was added to each reaction tube which was allowed to stand for 3min at room temperature. Supernatants were combined and centrifuged at 2500×g for 5min at 4°C. The pellet resuspended in 2mL of DMEM-F12 was used as a crude testicular interstitial cell suspension, which was subject to discontinuous Percoll gradients for further purification. Two millilitre of 75% Percoll gradient was added to the centrifuge tube. Above this layer, 70%, 58%, 30% and 5% gradients of Percoll were laid gently one over the other. 2mL of crude Leydig cell suspension was then applied on top of this discontinuous gradient and centrifuged at 3000×g for 30min at 4°C. After centrifugation, most of Leydig cells were observed in 30% Percoll gradients. Leydig cells were transferred to centrifuge tube containing DMEM-F12 medium. After mixing, the tube was centrifuged at 2500×g for 10min at 4°C. Cell purity was assessed by histochemical localization analysis of 3β-HSD according to Aldred & Cooke [26]. Leydig cells were incubated with to DMEM-F12 supplemented with 10% fetal bovine serum (FBS), 5mg/mL transferrin, 2mM L-glutamine, 1.75mM HEPES, 100IU/mL penicillin and 100mg/mL streptomycin, and cultured in an atmosphere of 95% air and 5% CO_2_ at 37°C.

### Detection of testosterone content by radioimmunoassay (RIA)

The concentration of testosterone in primary Leydig cells under basal or stimulated conditions was determined with RIA kit. After culturing for 24 h in DMEM-F12 medium at 37°C, cells were incubated with DHEA (0μM, 1μM, 50μM or 100μM) for 24 h, partly cells was collected and other cells was exposed to 300μM H_2_O_2_ for 8h. After incubation, cells were disrupted ultrasonically on ice and centrifuged at 2500×g for 10min at 4°C. The testosterone concentrations in the supernatants were determined (intra-variation coefficients < 10%, inter-variation coefficients < 15%).

### Cell viability assay

Cells were seeded on a 96-well plate (1×10^5^ cells/well) and treated with 0, 1, 10, 50 or 100μM DHEA for 24h, then exposed to 300μM H_2_O_2_ for another 8h before addition of MTT solution. H_2_O_2_-free control cultures received an equal volume of dimethyl sulfoxide (DMSO, not exceeding 0.1%). 20μL of 5mg/mL MTT (3- [4, 5-dimethylthiazol-2-yl]-2, 5 diphenyl tetrazolium bromide) were added to each well. After 4h of culture, the culture medium was removed and the blue formazan crystals that had formed were dissolved in 50μL DMSO. The optical density of the formazan generated from MTT was measured at 490nm using a Microplate reader (Bio-Rad, USA).

### Measurement of antioxidant parameters

Cells were grown in 6-well plates (1×10^5^ cells/well) and treated with 0, 1, 10, 50 or 100μM DHEA for 24h, and then exposed to 300μM H_2_O_2_ for 8h. After incubation, the cells were then harvested, disrupted ultrasonically in ice and centrifuged at 2500×g for 10min at 4°C. The supernatants were collected and stored -20°C for subsequent analysis. The superoxide dismutase (SOD), glutathione peroxidase (GSH-Px), catalase (CAT), peroxidase (POD) activities and malondialdehyde (MDA) contents were determined using commercially available assay kits (Jiancheng Bioengineering Institute, China) following the manufacturers’ protocols.

### Reactive oxygen species content detected

ROS and O_2_^-^ content were measured with the fluoroprobe 2′,7′-dichlorodihydrofluorescein diacetate (H2DCF-DA) and dihydroethidium (DHE), as previously described [27]. Briefly, cells were incubated with DHEA (0, 1, 10, 50 or 100μM) for 24h and then exposed to 300μM H_2_O_2_ for 8h. After incubation, cells were washed and incubated with 5mM H2DCF-DA or 2mM DHE for 30min at 37°C. The cells were washed and collected by centrifugation and suspended in PBS. Fluorescent intensity was measured by a FACSCalibur™ flow cytometry (Becton Dickinson, USA). Hydroxyl radical (·OH) contents were determined spectrophotometrically using commercially available assay kit (Biyuntian Bioengineering Institute, China) following the manufacturer’s protocols.

### Assessment of DNA damage by the alkaline comet assay

DNA damage in Leydig cells was evaluated using a Trevigen Comet AssayTM kit (Trevigen Inc., USA) according to the manufacturer’s instructions. Briefly, cells were incubated with DHEA (0, 1, 10, 50 or 100μM) for 24h and then exposed to 300μM H_2_O_2_ for 8h. After incubation, cells were washed and suspended in cold PBS at 1×10^5^ cells/mL. 50μL cells suspension and 500μL low-melting agarose (1%) were mixed and spread onto the comet slide, another layer of 1% agarose gel was then added. The slide was immersed in lysis buffer (2.5M NaCl, 100mM EDTA, 10mM Tris-base, 1% sodium lauryl sarcosinate, 1% Triton X-100, pH10) for at least 60 min at 4°C. Denaturation was performed in an alkali solution (300mM NaOH, 1mM EDTA, pH13.5) for 20min in the dark. The slide was then transferred to fresh alkaline (300mM NaOH, 1mM EDTA, pH13.5) and subjected to electrophoresis at 1V/cm, 300mA for 40min in darkness at 4°C. Thereafter, the slides were washed with neutralization buffer (0.4M Tris-HCl, pH7.4) and immersed in ice cold 100% ethanol for 5min and air-dried. DNA was stained with 50mL of SYBR dye for 20min in the refrigerator and immediately analyzed using a Nikon epifluorescence microscope. For each slide, 50 randomly chosen comets were analyzed using a Nikon epifluorescence microscope. Fluorescent images of single cells were captured and computed for comet parameters: comet length, tail moment, olive tail moment, head DNA and tail DNA, using the Biolab Comet v1.0 image analysis software.

### Annexin V/propidium iondine staining assay

Cells undergoing apoptosis were determined using Annexin V-FITC and propidium iodine (PI) dual staining and measured by flow cytometry. Briefly, cells were incubated with DHEA (0, 1, 10, 50 or 100μM) for 24h and then exposed to 300μM H_2_O_2_ for 8h. After incubation, the cells were harvested and washed with cold phosphate buffered saline. 200μL of Annexin V-FITC stock solution was added to the cells and incubation continued for 30min at 4°C in the dark. This was followed by a further incubation with the propidium iodide solution (10μL, containing RNase). The cells were then immediately detected by FACSCalibur™ flow cytometry (Becton Dickinson, USA) to measure the cell apoptosis. Approximately 10,000 cells were analyzed in each sample.

### Assay of apoptosis factors mRNA level by Real-time quantitative PCR

After treatment, total RNA was extracted from cells using the Trizol reagent (TaKaRa, Japan), according to the manufacturer’s protocol. The RNA concentration was determined by measuring the absorbance at 260nm (Eppendorf Biophotometer, Germany). 2μg of total RNA were reverse transcribed by incubation for 1h at 37°C in a 25μL mixture comprising of 100U M-KGV reverse transcriptase, 8U RNase inhibitor, 0.5μg of oligo (dT), 50mM Tris-HCl (pH 8.3), 3mM MgCl2, 75mM KCl, 10mM DDT and 0.8mM dNTP. An aliquot of cDNA sample was mixed with 25μL SYBR Green PCR Master Mix (TaKaRa, Japan), in the presence of 10pmol of forward and reverse primer for *β-actin* (use as an internal control), *Bax*, *Bcl-2*, *capase-9* and *capase-3* (Table 1). All samples were analyzed in duplicate using an ABI Prism 7300 Sequence Detection System (Applied Biosystems, Sweden) using a program of 95°C for 1min, followed by 35 cycles 95°C for 30s, 60°C for 30s and 72°C for 20s. Fold changes were calculated using the 2^-ΔΔCT^ method. The primers were designed using Primes Premier 5 and synthesized by Takara Biotechnology Co. Ltd (Dalian, China).

**Table 1:** Prime sequence of β-actin and targeted gene

Gene	Genebank accession Number	Primer sequences(5′-3′)	Orientation	Product size(bp)
β-actin	NM_031144	CCCTGTGCTGCTCACCGA ACAGTGTGGGTGACCCCGTC	Forward Reverse	186
Bax	NM_007527	GCAGGGAGGATGGCTGGGGAGA TCCAGACAAGCAGCCGCTCACG	Forward Reverse	352
Bcl-2	NM_016993	CGACTTTGCAGAGATGTCCA CATCCACAGAGCGATGTTGT	Forward Reverse	202
Caspase-9	NM_031632	GCCTCATCATCATCAACAACG TCTACGACAGGGTGGTC	Forward Reverse	283
Caspase-3	NM_012922	CTGGACTGCGGTATTGAG CGAATGAGATGGCGTGGG	Forward Reverse	342

### Statistical analyses

Data were expressed as means ± SD and differences were considered significant at *P < 0.05*. The effect of DHEA on testosterone and the cell viability induced by H_2_O_2_ were analyzed by *t*-test. Other data were analyzed with one-way ANOVA and treatment differences were subjected to a Duncan’s multiple comparison tests. All statistical analyses were performed with SPSS 13.0 for Windows (StatSoft, Inc., Tulsa, OK, USA).
